# Supplementary material for: The Effects of In-Plane Spatial Resolution on CT-Based Radiomic Features’ Stability with and without ComBat Harmonization
Source: Cancers (Basel). 2021 Apr 13;13(8):1848. doi: 10.3390/cancers13081848 (PMC8103509; doi:10.3390/cancers13081848)
Supplement: Supplementary file 1 [file cancers-13-01848-s001.zip › cancers-1179116-supplementary.docx]

The Effects of In-Plane Spatial Resolution on CT-Based Radiomic Features’ Stability with and without ComBat Harmonization

Abdalla Ibrahim ^1,2,3,4,^*, Turkey Refaee ^1,5,†^, Sergey Primakov ^1,4,†^, Bruno Barufaldi ^6^, Raymond J. Acciavatti ^6^, Renée W. Y. Granzier ^7^, Roland Hustinx ^3^, Felix M. Mottaghy ^2,4^, Henry C. Woodruff ^1,2^, Joachim E. Wildberger ^2^, Philippe Lambin ^1,2^ and Andrew D. A. Maidment ^6^

^1^ The D-Lab, Department of Precision Medicine, GROW—School for Oncology, Maastricht University, 6200 Maastricht, The Netherlands; t.refaee@maastrichtuniversity.nl (T.R.); S.primakov@maastrichtuniversity.nl (S.P.); h.woodruff@maastrichtuniversity.nl (H.C.W.); philippe.lambin@maastrichtuniversity.nl (P.L.)

^2^ Department of Radiology and Nuclear Medicine, Maastricht University Medical Centre+, 6200 Maastricht, The Netherlands; felix.mottaghy@mumc.nl (F.M.M.); j.wildberger@mumc.nl (J.E.W.)

^3^ Division of Nuclear Medicine and Oncological Imaging, Department of Medical Physics, University Hospital of Liège and GIGA CRC-in vivo imaging, University of Liège, 4000 Liege, Belgium; rhustinx@ulg.ac.be (R.H.)

^4^ Department of Nuclear Medicine and Comprehensive diagnostic center Aachen (CDCA), University Hospital RWTH Aachen University, 52074 Aachen, Germany

^5^ Department of Diagnostic Radiology, Faculty of Applied Medical Sciences, Jazan University, 45142 Jazan, Saudi Arabia

^6^ Department of Radiology, Perelman School of Medicine, University of Pennsylvania, Philadelphia, PA 19104, USA; Bruno.Barufaldi@pennmedicine.upenn.edu (B.B.); racci@pennmedicine.upenn.edu (R.J.A.); Andrew.Maidment@pennmedicine.upenn.edu (A.D.A.M.)

^7^ Department of Surgery, GROW—School for Oncology, Maastricht University Medical Centre+, 6200 Maastricht, the Netherlands; r.granzier@maastrichtuniversity.nl

***** Correspondence: a.ibrahim@maastrichtuniversity.nl

† Authors contributed equally.


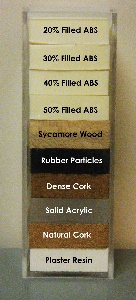


**Figure S1.** The scanned CCR Phantom.


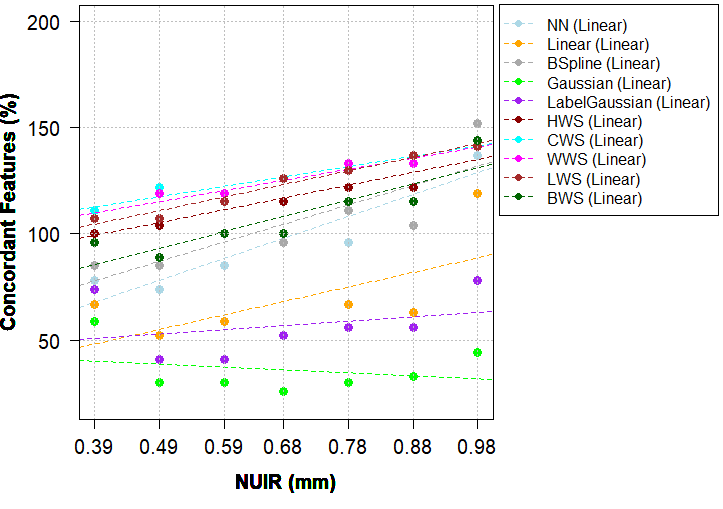


**Figure S2.** The percentage of concordant features following resampling compared to no resampling with linear trendlines, LightSpeed model.


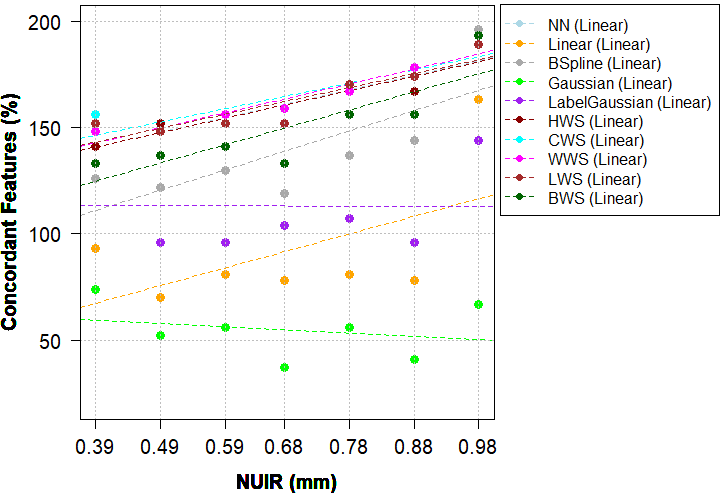


**Figure S3.** The percentage of concordant features following resampling and ComBat harmonization compared to no resampling with linear trendlines, LightSpeed Pro 32 model.

| **Table S1.** Number of pair-wise concordant HRFs with a CCC > 0.9 before resampling, LightSpeed Pro 32 model. | | | | | | |
| --- | --- | --- | --- | --- | --- | --- |
| **Scan** | **CCR-2-022** | **CCR-2-023** | **CCR-2-024** | **CCR-2-025** | **CCR-2-026** | **CCR-2-027** |
| **CCR-2-023** | 72 (79.1%) |  |  |  |  |  |
| **CCR-2-024** | 60 (65.9%) | 82 (90.1%) |  |  |  |  |
| **CCR-2-025** | 46 (50.5%) | 50 (54.9%) | 62 (68.1%) |  |  |  |
| **CCR-2-026** | 52 (57.1%) | 62 (68.1%) | 74 (81.3%) | 67 (73.6%) |  |  |
| **CCR-2-027** | 39 (42.8%) | 44 (48.3%) | 51 (56.0%) | 67 (73.6%) | 66 (72.5%) |  |
| **CCR-2-028** | 41 (45.0%) | 44 (48.3%) | 49 (53.8%) | 43 (47.2%) | 68 (74.7%) | 67 (73.6%) |

| Table S2. Number of pair-wise concordant HRFs with a CCC > 0.9 after ComBat, LightSpeed Pro 32 model. | | | | | | |
| --- | --- | --- | --- | --- | --- | --- |
| Scan | **CCR-022** | **CCR-023** | **CCR-024** | **CCR-025** | **CCR-026** | **CCR-027** |
| CCR-023 | 76 (83.5%) |  |  |  |  |  |
| CCR-024 | 67 (73.6%) | 83 (91.2%) |  |  |  |  |
| CCR-025 | 56 (61.5%) | 60 (65.9%) | 68 (74.7%) |  |  |  |
| CCR-026 | 60 (65.9%) | 69 (75.8%) | 75 (82.4%) | 70 (76.9%) |  |  |
| CCR-027 | 53 (58.2%) | 54 (59.3%) | 58 (63.7%) | 70 (76.9%) | 69 (75.8%) |  |
| CCR-028 | 49 (53.8%) | 52 (57.1%) | 57 (62.6%) | 55 (60.4%) | 72 (79.1%) | 70 (76.9%) |

| Table S3. Number of pair-wise concordant HRFs with a CCC > 0.9 after resampling* using CWS, LightSpeed Pro 32 model. | | | | | | |
| --- | --- | --- | --- | --- | --- | --- |
| Scan | **CCR-022** | **CCR-023** | **CCR-024** | **CCR-025** | **CCR-026** | **CCR-027** |
| CCR-023 | 90 (98.9%) |  |  |  |  |  |
| CCR-024 | 89 (97.8%) | 90 (98.9%) |  |  |  |  |
| CCR-025 | 74 (81.3%) | 75 (82.4%) | 75 (82.4%) |  |  |  |
| CCR-026 | 88 (96.7%) | 90 (98.9%) | 90 (98.9%) | 74 (81.3%) |  |  |
| CCR-027 | 70 (76.9%) | 71 (78.0%) | 70 (76.9%) | 69 (75.8%) | 70 (76.9%) |  |
| CCR-028 | 52 (57.1%) | 51 (56.0%) | 51 (56.0%) | 38 (41.7%) | 64 (70.3%) | 62 (68.1%) |
| * All scans were resampled to the median pixel spacing value (0.49*0.49 mm^2^). | | | | | | |

| Table S4. Number of pair-wise concordant HRFs with a CCC > 0.9 after ComBat following resampling* using CWS, LightSpeed Pro 32 model. | | | | | | |
| --- | --- | --- | --- | --- | --- | --- |
| Scan | **CCR-022** | **CCR-023** | **CCR-024** | **CCR-025** | **CCR-026** | **CCR-027** |
| CCR-023 | 90 (98.9%) |  |  |  |  |  |
| CCR-024 | 89 (97.8%) | 90 (98.9%) |  |  |  |  |
| CCR-025 | 76 (83.5%) | 76 (83.5%) | 75 (82.4%) |  |  |  |
| CCR-026 | 88 (96.7%) | 90 (98.9%) | 90 (98.9%) | 77 (84.6%) |  |  |
| CCR-027 | 72 (79.1%) | 73 (80.2%) | 73 (80.2%) | 71 (78.0%) | 73 (80.2%) |  |
| CCR-028 | 61 (67.0%) | 60 (65.9%) | 62 (68.1%) | 46 (50.5%) | 69 (75.8%) | 68 (74.7%) |
| * All scans were resampled to the median pixel spacing value (0.49*0.49 mm^2^). | | | | | | |

| Table S5. Summary of the number of concordant features (CCC>0.9) before and after resampling, Discovery STE model. | | | | | | | | | | |
| --- | --- | --- | --- | --- | --- | --- | --- | --- | --- | --- |
|  | **NN** | **Linear** | **BSpline** | **Gaussian** | **LabelGaussian** | **HWS** | **CWS** | **WWS** | **LWS** | **BWS** |
| ***Pixel Spacing [0.39x0.39]** |  |  |  |  |  |  |  |  |  |  |
| CCC>0.9 only after | 5 | 6 | 12 | 1 | 3 | 11 | 15 | 14 | 15 | 14 |
| CCC>0.9 before and after | 24 | 15 | 26 | 6 | 17 | 31 | 32 | 32 | 32 | 27 |
| CCC>0.9 only before | 12 | 21 | 10 | 30 | 19 | 5 | 4 | 4 | 4 | 9 |
| **Overall**** | **80.6%** | **58.3%** | **105.6%** | **19.4%** | **55.6%** | **116.7%** | **130.6%** | **127.8%** | **130.6%** | **113.9%** |
| **Pixel Spacing [0.49x0.49]** |  |  |  |  |  |  |  |  |  |  |
| CCC>0.9 only after | 7 | 3 | 14 | 1 | 3 | 15 | 16 | 16 | 16 | 15 |
| CCC>0.9 before and after | 25 | 7 | 26 | 6 | 17 | 32 | 32 | 32 | 32 | 28 |
| CCC>0.9 only before | 11 | 29 | 10 | 30 | 19 | 4 | 4 | 4 | 4 | 8 |
| **Overall** | **88.9%** | **27.8%** | **111.1%** | **19.4%** | **55.6%** | **130.6%** | **133.3%** | **133.3%** | **133.3%** | **119.4%** |
| **Pixel Spacing [0.59x0.59]** |  |  |  |  |  |  |  |  |  |  |
| CCC>0.9 only after | 10 | 7 | 15 | 1 | 3 | 17 | 17 | 17 | 16 | 15 |
| CCC>0.9 before and after | 26 | 15 | 27 | 6 | 18 | 32 | 32 | 33 | 32 | 29 |
| CCC>0.9 only before | 10 | 21 | 9 | 30 | 18 | 4 | 4 | 3 | 4 | 7 |
| **Overall** | **100.0%** | **61.1%** | **116.7%** | **19.4%** | **58.3%** | **136.1%** | **136.1%** | **138.9%** | **133.3%** | **122.2%** |
| **Pixel Spacing [0.68x0.68]** |  |  |  |  |  |  |  |  |  |  |
| CCC>0.9 only after | 12 | 6 | 14 | 1 | 3 | 17 | 15 | 16 | 15 | 15 |
| CCC>0.9 before and after | 27 | 14 | 26 | 7 | 17 | 32 | 33 | 33 | 32 | 30 |
| CCC>0.9 only before | 9 | 22 | 10 | 29 | 19 | 4 | 3 | 3 | 4 | 6 |
| **Overall** | **108.3%** | **55.6%** | **111.1%** | **22.2%** | **55.6%** | **136.1%** | **133.3%** | **136.1%** | **130.6%** | **125.0%** |
| **Pixel Spacing [0.78x0.78]** |  |  |  |  |  |  |  |  |  |  |
| CCC>0.9 only after | 10 | 6 | 17 | 1 | 3 | 18 | 17 | 17 | 17 | 17 |
| CCC>0.9 before and after | 26 | 10 | 30 | 6 | 20 | 33 | 33 | 33 | 33 | 31 |
| CCC>0.9 only before | 10 | 26 | 6 | 30 | 16 | 3 | 3 | 3 | 3 | 5 |
| **Overall** | **100.0%** | **44.4%** | **130.6%** | **19.4%** | **63.9%** | **141.7%** | **138.9%** | **138.9%** | **138.9%** | **133.3%** |
| **Pixel Spacing [0.88x0.88]** |  |  |  |  |  |  |  |  |  |  |
| CCC>0.9 only after | 14 | 7 | 17 | 1 | 3 | 19 | 18 | 18 | 18 | 17 |
| CCC>0.9 before and after | 32 | 17 | 31 | 7 | 21 | 35 | 34 | 35 | 33 | 32 |
| CCC>0.9 only before | 4 | 19 | 5 | 29 | 15 | 1 | 2 | 1 | 3 | 4 |
| **Overall** | **127.8%** | **66.7%** | **133.3%** | **22.2%** | **66.7%** | **150.0%** | **144.4%** | **147.2%** | **141.7%** | **136.1%** |
| **Pixel Spacing [0.98x0.98]** |  |  |  |  |  |  |  |  |  |  |
| CCC>0.9 only after | 19 | 15 | 19 | 1 | 4 | 20 | 19 | 19 | 19 | 20 |
| CCC>0.9 before and after | 34 | 34 | 35 | 7 | 25 | 35 | 35 | 35 | 35 | 35 |
| CCC>0.9 only before | 2 | 2 | 1 | 29 | 11 | 1 | 1 | 1 | 1 | 1 |
| **Overall** | **147.2%** | **136.1%** | **150.0%** | **22.2%** | **80.6%** | **152.8%** | **150.0%** | **150.0%** | **150.0%** | **152.8%** |
| * The NUIR value  ** Overall is calculated by dividing the number of features concordant after resampling divided by the number of concordant features before resampling. | | | | | | | | | | |

| **Table S6.** Summary of the number of concordant features (CCC>0.9) before and after resampling, LightSpeed Pro 32 model. | | | | | | | | | | |
| --- | --- | --- | --- | --- | --- | --- | --- | --- | --- | --- |
|  | **NN** | **Linear** | **BSpline** | **Gaussian** | **LabelGaussian** | **HWS** | **CWS** | **WWS** | **LWS** | **BWS** |
| ***Pixel Spacing [0.39x0.39]** |  |  |  |  |  |  |  |  |  |  |
| CCC>0.9 only after | 3 | 3 | 4 | 4 | 4 | 6 | 8 | 7 | 7 | 5 |
| CCC>0.9 before and after | 18 | 15 | 19 | 12 | 16 | 21 | 22 | 22 | 22 | 21 |
| CCC>0.9 only before | 9 | 12 | 8 | 15 | 11 | 6 | 5 | 5 | 5 | 6 |
| **Overall**** | **77.8%** | **66.7%** | **85.2%** | **59.3%** | **74.1%** | **100.0%** | **111.1%** | **107.4%** | **107.4%** | **96.3%** |
| **Pixel Spacing [0.49x0.49]** |  |  |  |  |  |  |  |  |  |  |
| CCC>0.9 only after | 3 | 1 | 3 | 1 | 3 | 6 | 7 | 7 | 6 | 4 |
| CCC>0.9 before and after | 17 | 13 | 20 | 7 | 8 | 22 | 26 | 25 | 23 | 20 |
| CCC>0.9 only before | 10 | 14 | 7 | 20 | 19 | 5 | 1 | 2 | 4 | 7 |
| **Overall** | **74.1%** | **51.9%** | **85.2%** | **29.6%** | **40.7%** | **103.7%** | **122.2%** | **118.5%** | **107.4%** | **88.9%** |
| **Pixel Spacing [0.59x0.59]** |  |  |  |  |  |  |  |  |  |  |
| CCC>0.9 only after | 5 | 2 | 5 | 1 | 3 | 7 | 7 | 7 | 7 | 5 |
| CCC>0.9 before and after | 18 | 14 | 22 | 7 | 8 | 24 | 25 | 25 | 24 | 22 |
| CCC>0.9 only before | 9 | 13 | 5 | 20 | 19 | 3 | 2 | 2 | 3 | 5 |
| **Overall** | **85.2%** | **59.3%** | **100.0%** | **29.6%** | **40.7%** | **114.8%** | **118.5%** | **118.5%** | **114.8%** | **100.0%** |
| **Pixel Spacing [0.68x0.68]** |  |  |  |  |  |  |  |  |  |  |
| CCC>0.9 only after | 7 | 1 | 5 | 1 | 5 | 8 | 8 | 8 | 8 | 6 |
| CCC>0.9 before and after | 19 | 13 | 21 | 6 | 9 | 23 | 26 | 26 | 26 | 21 |
| CCC>0.9 only before | 8 | 14 | 6 | 21 | 18 | 4 | 1 | 1 | 1 | 6 |
| **Overall** | **96.3%** | **51.9%** | **96.3%** | **25.9%** | **51.9%** | **114.8%** | **125.9%** | **125.9%** | **125.9%** | **100.0%** |
| **Pixel Spacing [0.78x0.78]** |  |  |  |  |  |  |  |  |  |  |
| CCC>0.9 only after | 6 | 3 | 8 | 1 | 6 | 9 | 10 | 10 | 9 | 8 |
| CCC>0.9 before and after | 20 | 15 | 22 | 7 | 9 | 24 | 26 | 26 | 26 | 23 |
| CCC>0.9 only before | 7 | 12 | 5 | 20 | 18 | 3 | 1 | 1 | 1 | 4 |
| **Overall** | **96.3%** | **66.7%** | **111.1%** | **29.6%** | **55.6%** | **122.2%** | **133.3%** | **133.3%** | **129.6%** | **114.8%** |
| **Pixel Spacing [0.88x0.88]** |  |  |  |  |  |  |  |  |  |  |
| CCC>0.9 only after | 10 | 3 | 5 | 2 | 7 | 9 | 11 | 10 | 11 | 7 |
| CCC>0.9 before and after | 23 | 14 | 23 | 7 | 8 | 24 | 26 | 26 | 26 | 24 |
| CCC>0.9 only before | 4 | 13 | 4 | 20 | 19 | 3 | 1 | 1 | 1 | 3 |
| **Overall** | **122.2%** | **63.0%** | **103.7%** | **33.3%** | **55.6%** | **122.2%** | **137.0%** | **133.3%** | **137.0%** | **114.8%** |
| **Pixel Spacing [0.98x0.98]** |  |  |  |  |  |  |  |  |  |  |
| CCC>0.9 only after | 12 | 11 | 14 | 2 | 7 | 11 | 11 | 11 | 11 | 12 |
| CCC>0.9 before and after | 25 | 21 | 27 | 10 | 14 | 27 | 27 | 27 | 27 | 27 |
| CCC>0.9 only before | 2 | 6 | 0 | 17 | 13 | 0 | 0 | 0 | 0 | 0 |
| **Overall** | **137.0%** | **118.5%** | **151.9%** | **44.4%** | **77.8%** | **140.7%** | **140.7%** | **140.7%** | **140.7%** | **144.4%** |
| * The NUIR value  ** Overall is calculated by dividing the number of features concordant after resampling divided by the number of concordant features before resampling. | | | | | | | | | | |

**List S1:** HRFs with CCC>0.9 across all pairs on Discovery STE model.

1. original_firstorder_90Percentile
2. original_firstorder_Entropy
3. original_firstorder_Maximum
4. original_firstorder_Mean
5. original_firstorder_Median
6. original_firstorder_Minimum
7. original_firstorder_Range
8. original_firstorder_RootMeanSquared
9. original_firstorder_TotalEnergy
10. original_firstorder_Uniformity
11. original_glcm_Autocorrelation
12. original_glcm_DifferenceEntropy
13. original_glcm_Id
14. original_glcm_Idm
15. original_glcm_JointAverage
16. original_glcm_JointEnergy
17. original_glcm_JointEntropy
18. original_glcm_MaximumProbability
19. original_gldm_HighGrayLevelEmphasis
20. original_gldm_LargeDependenceEmphasis
21. original_gldm_LargeDependenceLowGrayLevelEmphasis
22. original_gldm_LowGrayLevelEmphasis
23. original_gldm_SmallDependenceEmphasis
24. original_gldm_SmallDependenceHighGrayLevelEmphasis
25. original_glrlm_GrayLevelNonUniformityNormalized
26. original_glrlm_HighGrayLevelRunEmphasis
27. original_glrlm_RunLengthNonUniformityNormalized
28. original_glrlm_RunPercentage
29. original_glrlm_ShortRunEmphasis
30. original_glrlm_ShortRunHighGrayLevelEmphasis
31. original_glszm_GrayLevelNonUniformityNormalized
32. original_glszm_GrayLevelVariance
33. original_glszm_HighGrayLevelZoneEmphasis
34. original_glszm_SmallAreaHighGrayLevelEmphasis
35. original_glszm_ZonePercentage
36. original_ngtdm_Complexity

**List S2:** HRFs with CCC>0.9 across all pairs on LighSpeed Pro 32 model.

1. original_firstorder_90Percentile
2. original_firstorder_Entropy
3. original_firstorder_Maximum
4. original_firstorder_Mean
5. original_firstorder_Median
6. original_firstorder_Minimum
7. original_firstorder_Range
8. original_firstorder_RootMeanSquared
9. original_firstorder_TotalEnergy
10. original_firstorder_Uniformity
11. original_glcm_Autocorrelation
12. original_glcm_ClusterProminence
13. original_glcm_DifferenceEntropy
14. original_glcm_Id
15. original_glcm_Idm
16. original_glcm_JointEntropy
17. original_glcm_MaximumProbability
18. original_gldm_HighGrayLevelEmphasis
19. original_gldm_LargeDependenceEmphasis
20. original_glrlm_HighGrayLevelRunEmphasis
21. original_glrlm_RunLengthNonUniformityNormalized
22. original_glrlm_RunPercentage
23. original_glrlm_ShortRunEmphasis
24. original_glrlm_ShortRunHighGrayLevelEmphasis
25. original_glszm_GrayLevelVariance
26. original_glszm_HighGrayLevelZoneEmphasis
27. original_glszm_SmallAreaHighGrayLevelEmphasis

**List S3:** Non-highly correlated HRFs with CCC>0.9 across all pairs on Discovery STE model.

1. original_firstorder_Maximum
2. original_firstorder_Median
3. original_firstorder_Minimum
4. original_firstorder_Range
5. original_glcm_MaximumProbability
6. original_gldm_LowGrayLevelEmphasis
7. original_glrlm_GrayLevelNonUniformityNormalized
8. original_glszm_SmallAreaHighGrayLevelEmphasis
9. original_ngtdm_Complexity

**List S4:** Non-highly correlated HRFs with CCC>0.9 across all pairs on LighSpeed Pro 32 model.

1. original_firstorder_90Percentile
2. original_firstorder_Maximum
3. original_firstorder_Minimum
4. original_firstorder_Range
5. original_firstorder_Uniformity
6. original_glcm_Autocorrelation
7. original_glcm_ClusterProminence
8. original_gldm_LargeDependenceEmphasis
9. original_glszm_SmallAreaHighGrayLevelEmphasis
